# Variability in Global DNA Methylation Rate Across Tissues and Over Time in Sheep

**DOI:** 10.3389/fgene.2022.791283

**Published:** 2022-03-11

**Authors:** Laurence Drouilhet, Carole Moreno, Florence Plisson-Petit, Didier Marcon, Stéphane Fabre, Dominique Hazard

**Affiliations:** ^1^ GenPhySE, Université de Toulouse, INRAE, ENVT, Castanet-Tolosan, France; ^2^ P3R, UE 0332, INRAE, Osmoy, France

**Keywords:** sheep, epigenetics, methylation, blood, tissues

## Abstract

Recent studies highlighted the influence of epigenetic marks in the variability of many complex traits, both in plants and animals. These studied focused only on specific sites of the genome having differentially methylated profiles among individuals and/or tissues. In contrast, we recently used the methylation rate of the entire genome as a unique measure considered as a novel quantitative phenotype in sheep. This phenotype named global DNA methylation rate (GDMR), measured by luminometric assay, integrates the methylation level of each CpG dinucleotide within the 6 million of CCGG sites along the ovine genome. GDMR measured in blood previously showed moderate heritability of 0.20 and provided evidence for a genetic determinism. The main objective of the present study was to better characterize the GDMR phenotype in various tissues and investigate its variability in several breeds of sheep reared in the same environment. GDMR was measured on blood samples collected monthly from 59 growing male and female lambs (24 Romane, 23 Blackbelly and 12 Charollais), between birth and 4 months of age. Blood GDMR was on average around 80% and was influenced by the sampling date (*p* < 0.001), the breed (*p* = 0.002) and the sex (*p* = 0.002). In addition, GDMR was determined in 12 somatic (frontal lobe, pituitary gland, heart, lung, sub cutaneous and perirenal adipose tissue, skeletal muscle, liver, spleen, adrenal gland, medulla and cortical kidney) and 6 reproductive tissues (ovary, oviduct, uterus, testis, epididymis and seminal vesicle). GDMR was on average 70% in somatic tissues but marked variation was observed depending on the tissue. The GDMR measured in blood was higher than that measured in other somatic tissues, and is not a good proxy of less accessible tissues. Female reproductive tissues had a 10% higher GDMR than male reproductive tissues. We demonstrated a significant influence of the breed on blood GDMR, certainly reflecting the influence of different genetic backgrounds. The effect of the breed on GDMR may be related to their specific abilities to adapt to and live in different conditions.

## Introduction

Genetic improvement is widely used to increase animal production, health, and welfare in livestock systems. It is usually based on the use of phenotypes and pedigree data to estimate individual breeding values. DNA polymorphism data have recently started being used-depending on species and/or type of production-to estimate these values that characterize the landscape of genomic selection (Goddard et al., 2010). Variations in production traits are attributed to genetic determinism plus environment effects and their interactions. Recent knowledge on the effects of the environment focuses on epigenetic phenomenon. Variations of epigenetic marks influence the expression of genes and hence phenotypes in response to environmental conditions, demonstrating that the eukaryotic genome responds dynamically to changes in the environment to which all individuals are exposed (Goddard and Whitelaw, 2014). For example in rats, reduced maternal care immediately after birth can alter the epigenetic state and expression of the glucocorticoid receptor gene, resulting in them becoming stressed adults (Weaver et al., 2004). In plants, epigenetic recombinant inbred lines of *Arabidopsis thaliana*, obtained by an initial cross of isogenic parents with different DNA methylation profiles, have provided a powerful tool to investigate the role and significance of epigenetic alteration in almost identical genetic backgrounds (Johannes et al., 2009; Latzel et al., 2013). Using these model plant lines, DNA hypomethylation of the whole genome was associated with lower resistance to increased saline environment (Kooke et al., 2015). It was also shown that multiple DNA methylation changes induced across the genome can be stably inherited over at least eight generations in the absence of selection, and that these changes are associated with substantial heritable variation in two complex traits, flowering time and plant height (Johannes et al., 2009). Therefore, new scientific questions arise about the relationship between genetics and epigenetics as part of livestock species improvement and their adaptation to changing production systems (Goddard and Whitelaw, 2014). Until now, epigenetic modifications induced by diet have been studied in a variety of livestock species (for a review, see Murdoch et al., 2016). In Scottish blackface ewes, the extent of periconceptional availability of nutrients was linked to the methionine-folate cycle altered DNA methylation and body fat content of offspring (Sinclair et al., 2007). However, studies in mammals (laboratory animal models, human or livestock) focus either on the methylation status of few candidate gene loci or on whole genome differentially methylated sites depending on the physiological or pathological questions raised needed to address. Thus, even with the use of genome bisulfite sequencing, the global methylation level of genomic DNA is never considered as a differential parameter between experimental conditions or individuals. In a recent study, we focused on global DNA methylation rate (GDMR) as a novel quantitative phenotype measured by pyrosequencing luminometric methylation assay (LUMA) in sheep (Hazard et al., 2020).

The LUMA technology considers the CpG located at the CCGG site recognized by methylation-independent MspI and methylation-sensitive HapII restriction enzymes (Karimi et al., 2006a; Karimi et al., 2006b). *In silico*, we counted around 6 million of CCGG sites on the ovine reference genome, which gives a reliable approximation of the methylated cytosine for CG of the entire genome. We showed that inter-individual variability in GDMR in blood had an additive genetic component in sheep with moderate heritability (h^2^ = 0.20 ± 0.05). We then demonstrated the existence of genetic determinism of this particular epigenetic mark. Sheep are not only a species of agronomical interest but also represent an interesting animal model. Indeed, depending on the breed, sheep are reared in contrasted environments, sometimes in harsh conditions, thereby revealing their ability to adapt to and live in a wide range of conditions. Breeds of sheep also differ widely in their adaptive capacities depending on their origin and/or the production selection they undergo. Therefore, to better characterize the GDMR phenotype, we investigated variations in GDMR in the blood of several breeds of sheep reared in the same environment. We chose Romane (a stabilized composite breed between Romanov x Berrichon du Cher, Ricordeau et al., 1992) as representative of a rustic breed adapted to various temperate breeding conditions (intensive/indoors, extensive/outdoors), Charollais, a specialized breed for meat production (Huby et al., 2003) and Blackbelly as adapted to tropical environments with good resistance to parasitic gut worms (Sallé et al., 2012). The Romane and the Charollais breeds have common genetic components within the European northern sheep populations. However, among the French northern sheep populations, they appeared as the most divergent (Rochus et al., 2018). The Blackbelly breed originates from the Caribbean hair sheep breeds. Genome structural analysis of various sheep breed around the world clearly showed that Blackbelly breed is genetically different from the European breeds (Kijas et al., 2012; Spangler et al., 2017). In addition, to test whether the GDMR in the blood was representative of GDMR in other tissues, we searched for variations in GDMR in a large panel of ovine somatic and reproductive tissues.

## Material and Methods

### Ethics

The experiment was carried out in accordance with French national regulations for the use of animals for research purposes. The animals were bred at the INRAE experimental farm La Sapinière (Osmoy, France, experimental facility approval number C18–174–01). All the experiments were performed in accordance with the European Union Council directive (2010/63/UE) for the care and use of experimental animals for scientific purposes. Sampling procedures were approved by the local ethics committee C2EA-19 and authorized by the French Ministry of Higher Education, Research and Innovation (approval number: APAFIS#10243).

### Animals

In order to generate the experimental lambs, all the ewes (and thereafter lambs) from Romane, Blackbelly and Charolais breeds were reared at the same time within the same sheepfold of the experimental farm, but into separate pens according to breed. At the time of reproduction management, estrous cycle of ewes was synchronized by a male effect thanks to the presence of vasectomized males 15 days before the introduction of intact rams of the corresponding breed (two rams and 20 ewes per pen during 42 days). All ewes were managed the same manner according to their physiological status (before mating, gestation and suckling). We selected 49 least related experimental male and female lambs (24 Romane, 23 Blackbelly and 12 Charollais), born in September 2017, identified at birth with electronic ear tags and reared indoors with their dam in similar conditions until weaning (Supplementary Table S1). From 15 days of age, in addition to maternal suckling, lambs had access to concentrate feed. Lambs were weaned around 64 days of age. At weaning, animals had free access to concentrate feed plus straw. After reaching a weight of 35 kg, each animal was fed 700 gr of feed concentrate per day plus 1.3 kg of hay per day.

### Samples

Blood samples were collected once a month (in the morning) from each animal (*n* = 59) from birth to 4 months of age (*n* = 5 blood samples/animal). A complete blood count (CBC) was performed on each fresh blood sample by the *Centre de recherches biologiques* (ERBC, Baugy, France, https://www.erbc-group.com/). The measured parameters were white blood cell count (neutrophil, eosinophil, basophil, lymphocyte and monocyte), red blood cell count, red cell volume, reticulocytes, hemoglobin, hematocrit, mean corpuscular volume, mean corpuscular hemoglobin, mean corpuscular hemoglobin concentration, platelet count and platelet volume. Whole blood genomic DNA coming from all nucleated blood cells was extracted for each sample to determine the global DNA methylation rate.

A subset of 15 Romane and 15 Blackbelly lambs was slaughtered at 5 months of age (Supplementary Table S1). Blood and 12 somatic tissues (frontal lobe, pituitary gland, heart, lung, sub cutaneous adipose tissue, perirenal adipose tissue, muscle, liver, spleen, adrenal gland, medulla kidney and cortical kidney) were sampled from each animal. Three additional reproductive tissues, specific to each sex (female: ovary, oviduct, uterus; male: testis, epididymis and seminal vesicle), were also collected (Supplementary Table S2). The CBC of the blood sample collected at 5 months of age was not performed.

### LUminometric Methylation Assay Analyses

The global DNA methylation level of whole blood samples or tissues of individuals was measured using the LUMA assay (Karimi et al., 2006a; Karimi et al., 2006b). Genomic DNA was extracted from blood samples using a high-salt extraction method (Roussot et al., 2003) and from tissue samples using NucleoSpin Tissue kit (Macherey Nagel). DNA was digested by EcoRI + HpaII or EcoRI + MspI restriction enzymes (New England Biolabs) and then analyzed using a Q24 Pyromark sequencer (Qiagen). MspI and HpaII have the same recognition site (CCGG), whereas HpaII is inhibited by the presence of a 5-methylcytosine. EcoRI (recognition site: GAATTC) was used as internal control for normalization. Runs were analyzed with PyroMark Q24 1.0.10 software (Qiagen). The dispensation order for nucleotides was GTGTCACATGTGTG. Methylation levels were calculated from peak heights as [1 − [(HpaII(G)/EcoRI_Hpa(T))/(MspI(G)/EcoRI_Msp(T))] × 100]. The same control sample was used in each pyrosequencing run; its coefficient of variation calculated from 86 measurements was 1.4%.

### Statistical Analysis

The fixed effects of breed, sex, litter size (singleton, twice or triplet), date of sampling, CBC and tissue were tested, when relevant, by mixed linear models using the MIXED procedure of SAS software (SAS, 2008). Only the fixed effects and their interactions for which *p* < 0.05 were considered as significant and retained in the different models presented below.

For global DNA methylation rate of the blood and the complete blood count, the equation was:

$${\text{GDMR}}_{ijk}{\text{ or CBC}}_{ijk}=\mu +date\text{\_}sampling_{i}+breed_{j}+sex_{k}+a_{ij_{k}}+e_{ijk}\left(model1\right)$$ (1)

with *date_sampling*_*i*_ (5 levels), *breed*_*j*_ (3 levels) and *sex*_*k*_ (2 levels) as fixed effects, *a*_*ijk*_ the animal repeated across sampling (as random effect), and *e*_*ijk*_ the residual (as random effect). For the global DNA methylation rate of the blood considering the complete blood count, the equation was:

$${\text{GDMR}}_{ij}=\mu {+\text{CBC}}_{ij}+breed_{i}+sex_{j}+a_{ij}+e_{ij}\;\left(model2\right)$$ (2)

with *CBC*_*ij*_ as the covariable, with *breed*_*i*_ (3 levels) and *sex*_*j*_ (2 levels) as fixed effects, *a*_*ij*_ the animal repeated across sampling (as a random effect), and *e*_*ij*_ the residual (as a random effect). Model 3 was used to account for *GDMR* in tissues with the exception of tissues belonging to the reproductive tract*:*

$${\text{GDMR}}_{ij}=\mu {+\text{tissue}}_{i}+sex_{j}{+\text{tissue}}_{i}\text{*}sex_{j}+a_{ij}+e_{ij}\;\left(model3\right)$$ (3)

with tissue_*i*_ (13 levels), *sex*_*j*_ (2 levels), *a*_*ij*_ the animal repeated across tissues (as a random effect), and *e*_*ij*_ the residual (as a random effect). The effects of the age of the animal was tested but was not significant and therefore not retained in the model 3. Finally, for the GDMR of tissues belonging to the reproductive tract, the model 4 was:

$${\text{GDMR}}_{ij}=\mu +breed_{i}+tissue_{j}+a_{ij}+e_{ij}\;\left(model4\right)$$ (4)

with *breed*_*i*_ (2 levels), *tissue*_*j*_ (6 levels) and *a*_*ij*_ the animal repeated across tissues (as a random effect) and *e*_*ij*_ the residual (as a random effect).

Least squares means (LSMeans) for the fixed effects for each trait were obtained from these mixed linear models and were compared using Tukey test. LSMeans were considered significantly different with *p* < 0.05.

### Tissue-to-Tissue GDMR Correlation

Tissue-to-tissue GDMR correlations were estimated with the CORR procedure in SAS software (SAS, 2008), corresponding to Pearson correlation coefficients and the *p*-value associated.

## Results

### Global DNA Methylation Rate of Blood

**FIGURE 1:**
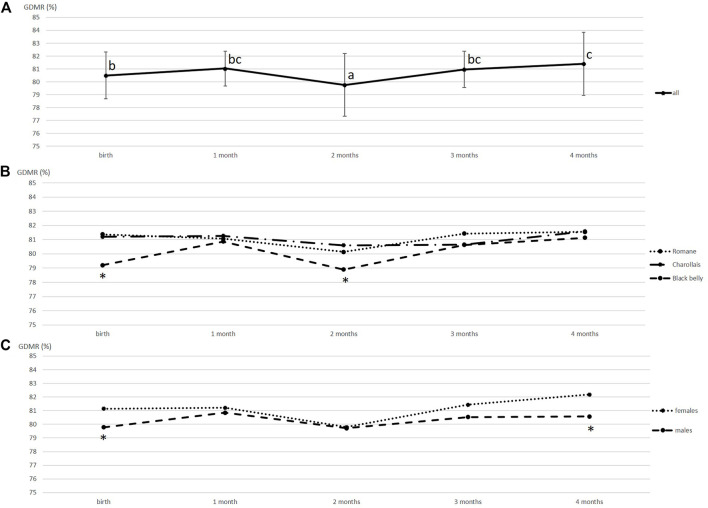
Variations in the global methylation rate of blood DNA over time. **(A)** GDMR means were calculated for 59 animals, vertical bars represent standard deviation, values with different superscripts (a, b and c) were found to differ significantly (*p* < 0.05). **(B)** GDMR means were calculated for each breed, Charollais (*n* = 12), Romane (*n* = 24) and Blackbelly (*n* = 23). For each date, asterisks indicate that the Blackbelly value differs significantly (*p* < 0.05). **(C)** GDMR means calculated according to sex, 28 males and 31 females. For each date, asterisks indicate that the male and female values differ significantly (*p* < 0.05).

GDMR was measured in blood samples collected once a month from 59 lambs of three different breeds. The sampling date, breed and sex were tested to explore GDMR variability using statistical model 1. The resulting *p* values are listed in Table 1. GDMR was significantly influenced by the sampling date (*p* < 0.001), breed (*p* = 0.002) and sex (*p* = 0.002). GDMR was lowest around 2 months of age (Figure 1A). Average GDMR over time (LSmeans) was lower in Blackbelly lambs (80.24%) than in Romane lambs (81.13%), GDMR in Charollais was between the two. The difference between breeds was particularly marked at birth (*p* = 0.02, Figure 1B) and at around 2 months of age (*p* = 0.05, Figure 1B). In addition, female lambs showed higher overall GDMR than males (81.12 vs. 80.37%; *p* = 0.002, Table 1). This difference is markedly visible at birth (*p* = 0.01, Figure 1C) and 4 months of age (*p* = 0.04, Figure 1C). However, no correlation was found between the different GDMR over time, whether considering the raw data or the residuals of the mixed model (data not shown).

**TABLE 1:** Effects of sampling date, breed, and sex on blood GDMR.

Effect	Level	LSMeans (SE)	*p*-value
Sampling date	Birth	80.50^b^ (0.24)	<0.0001
	1 month	81.05^bc^ (0.25)	
	2 months	79.76^a^ (0.25)	
	3 months	81.01^bc^ (0.25)	
	4 months	81.40^c^ (0.25)	
Breed	Romane	81.13^a^ (0.17)	0.002
	Blackbelly	80.24^b^ (0.18)	
	Charollais	80.87^ab^ (0.26)	
Sex	male	80.37^a^ (0.18)	0.002
	female	81.12^b^ (0.15)	

Statistical model 1 was used. LSMeans: Least square means, SE: standard error. For each effect, values with different superscripts (a, b, c) were found to differ significantly (p < 0.05).

### Variability of the Complete Blood Count and its Influence on GDMR

A CBC was performed on fresh blood from each sample. Among the five consecutive blood samples, on average, lymphocytes (60.4% ± 11.6) and neutrophils (31.2% ± 10.5) were the two most abundant cell types among the white blood cells (Table 2). For each cell type, we tested the effects of the sampling date, breed and sex (model 1). The associated *p*-values are listed in Table 3. All components of the CBC were significantly affected by the sampling date, and the breed effect was significant for all cell types except monocytes. The Blackbelly breed had more white blood cells than the Romane and Charollais breeds. Sex only affected the number of neutrophils (*p* = 0.03), which was higher in male than in female lambs.

**TABLE 2:** Proportion (%) of each nucleated cell type among the total white blood cells.

(%)	Lymphocytes	Neutrophils	Monocytes	Eosinophils	Basophils
µ	60.4	31.2	4.4	2.0	0.8
SD	11.6	10.5	2.8	1.6	0.3
min	4.9	7.4	0.2	0.3	0.2
max	84.8	72.3	34.6	10.3	2.5

µ: average; SD, standard deviation; min: minimum; max, maximum.

**TABLE 3:** Effects of the sampling date, breed and the sex on the complete blood count.

Blood component (Giga/L)	Effect			Breed LSMeans (SE)			Sex LSMeans (SE)	
	Sampling date	Breed	Sex	Romane	Blackbelly	Charollais	Male	Female
White blood cells	***	***	ns	8.13^a^ (0.28)	9.81^b^ (0.29)	8.00^a^ (0.40)	ns	ns
Lymphocytes	***	*	ns	4.90^a^ (0.23)	5.72^b^ (0.23)	5.19^ab^ (0.32)	ns	ns
Neutrophils	***	***	*	2.60^a^ (0.15)	3.26^b^ (0.15)	2.27^a^ (0.22)	2.93^a^ (0.15)	2.49^b^ (0.13)
Monocytes	***	ns	ns	ns	ns	ns	ns	ns
Eosinophils	**	***	ns	0.13^a^ (0.01)	0.25^b^ (0.01)	0.13^a^ (0.02)	ns	ns
Basophils	***	***	ns	0.06^a^ (0.004)	0.08^b^ (0.004)	0.06^a^ (0.006)	ns	ns

Statistical model 1 was used. ∗: *p* < 0.05, ∗∗: *p* < 0.01, ∗∗∗: *p* < 0.001. ns: not significant. Least square means (LSMeans) and the SE, of the breed and sex effects were estimated. For each effect, LSMeans, with different superscripts (a,b) were found to differ significantly (*p* < 0.05).

Next, we considered CBC as a covariable in order to explain GDMR variability (model 2). Among all CBC components, the number of neutrophils was the only significant parameter. It was therefore retained in the model. The Bayesian information criterion (BIC) was used to select the statistical model that best described our data (Table 4). Model 1 had the lowest BIC, indicating that given the sampling date effect, this model fitted the data better than the model with the neutrophil count.

**TABLE 4:** Comparison of the models for analyses of GDMR of the blood.

	Effect				Bayesian information criterion (BIC)
	Sampling date	Neutrophil count	Breed	Sex	
Model 1	<0.0001	/	0.002	0.002	1,182
Model 2	/	0.02	0.01	0.008	1,187

### Global DNA Methylation Rate of Somatic and Reproductive Tissues

Fifteen Blackbelly and 15 Romane lambs (representing 16 males and 14 females) were slaughtered at around 5 months of age to sample tissues. Samples of 13 different tissues (including a blood sample) were collected from both males and females. Three additional reproductive tissues, specific to each sex (testis, epididymis and seminal vesicle for males; ovary, oviduct and uterus for females), were also collected.

**FIGURE 2:**
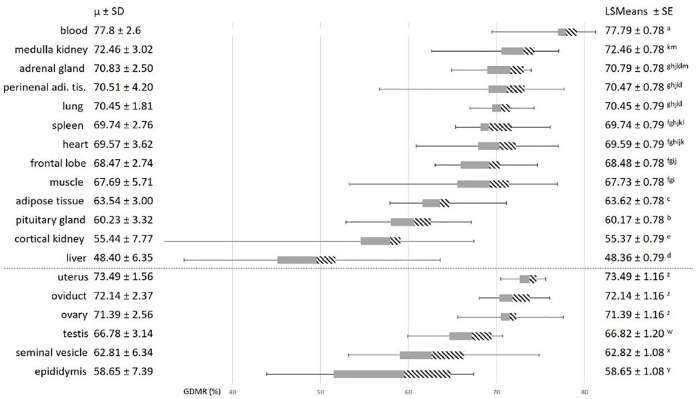
Variability in global DNA methylation rate in somatic and reproductive tissues. Average GDMR was calculated for 30 animals, 16 males and 14 females, (15 Blackbelly and 15 Romane sheep). The grey bars represent the second quartile, the dashed bars represent the third quartile, and the horizontal lines on each side correspond to the first (on the left) and the fourth (on the right) quartiles. The tissues below the dotted line belong to female and male reproductive tracts. Statistical model 3 was used for the analysis of somatic tissues. For each effect, values with different superscripts (a to m) were found to be significantly different (*p* < 0.05). Statistical model 4 was used for the analysis of reproductive tissues. For each effect, values with different superscripts (y to z) were found to differ significantly (*p* < 0.05). perirenal adi. tis.: perirenal adipose tissue.

**FIGURE 3:**
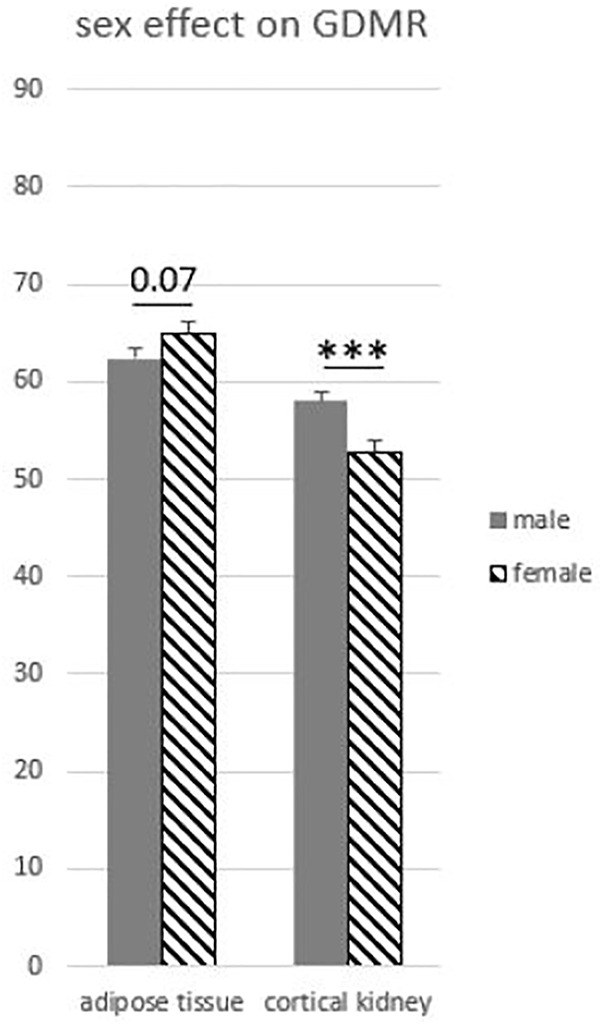
Global DNA methylation rate influenced by sex in adipose and kidney tissues. Model 3 was used for statistical analysis. ^∗∗∗^ *p* < 0.001.

To explore the variability of GDMR among the somatic tissues collected, we tested the effects of the tissue, age, sex, breed, and possible interactions. The age of the animal and the breed effects were not significant and were not retained in model 3. As expected, we observed a strong effect of the type of tissue on GDMR (*p* < 0.001; Figure 2). In particular, the GDMR of five tissues differed significantly from each other. The highest level of GDMR was measured in blood, while the lowest levels were measured in adipose tissue, pituitary gland, cortical kidney, and liver, and the levels of GDMR levels in liver being extremely low (Figure 2). The average GDMR of the other tissues ranged from 67.69 to 72.46%. Among somatic tissues, lung presented the lowest variability (70.45% ± 1.81) and cortical kidney the highest (55.44% ± 7.77). The overall interaction between sex and tissue was close to significance (*p* = 0.06). This could be explained by a tendency to an effect of sex in adipose tissue (*p* = 0.07), and a highly significant impact of sex on the cortical kidney (*p* = 0.0004). Indeed, the GDMR of cortical kidney was 9% lower in females than in males (Figure 3).

Focusing on the reproductive tract, we found a strong tissue effect (*p* < 0.0001, model 4). Interestingly, GDMR did not differ significantly between tissues in females and GDMR of female tissues was 10% higher than GDMR in male tissues (Figure 2). The GDMR of the female tissues was around 72% and quite homogenous (SD comprised between 1.56 and 2.56). In contrast, GDMR of male tissues was lower, ranging from 59 to 67% and more heterogeneous (SD comprised between 3.14 and 7.39) than that of females. Similar to the GDMR of blood, the overall GDMR of reproductive tissues was slightly lower in Blackbelly sheep than in Romane (breed effect, *p* = 0.03, model 4).

### Tissue-to-Tissue GDMR Correlation

At slaughter, using the GDMR dataset for 30 animals and 19 tissues (12 somatic tissues, three additional reproductive tissues specific to each sex and a blood sample), we looked for correlations of GDMR between tissues. The significant correlations ranged from −0.56 (*p* = 0.04) between ovary and adrenal gland, to 0.55 (*p* = 0.03) between medulla kidney and seminal vesicle (Supplementary Table S3). Using the blood previously sampled from birth to 4 months of age, the only significant strong correlation found was 0.63 between GDMR of ovary and GDMR of blood at 3 months of age (*p* = 0.02).

## Discussion

In mammals, DNA methylation occurs almost exclusively in the symmetric CG context and is estimated to occur at ∼70–80% of CG dinucleotides throughout the genome (Ehrlich et al., 1982). This estimation is consistent with the average GDMR of 70% found in the present study for somatic tissues in sheep. Mapping studies indicate that highly methylated sequences include satellite DNAs, repetitive elements (including transposons and their inert relics), non-repetitive intergenic DNA, and exons of genes. Unlike in plants, DNA methylation in mammals covers most of the genome, the main exception being CpG islands. Indeed, the most striking feature of vertebrate DNA methylation patterns is the presence of CpG islands, that is, unmethylated GC-rich regions that possess high relative densities of CpG and are positioned at the 5′ ends of many human genes (Suzuki and Bird, 2008). Computational analysis of the human genome sequence predicts 29,000 CpG islands (Lander et al., 2001; Venter et al., 2001). Local 5mC depletion is a reliable signature of promoters and enhancers: CpG sites in promoter-associated CpG islands are often less than 10% methylated, whereas distal regulatory sequences such as enhancers are commonly marked by levels of 5mC ranging from 10 to 50% (Stadler et al., 2011).

As the LUMA technology quantifies the methylation rate of CpG located in the CCGG restriction site, the entire genome is scanned independently of the features of the DNA (repeated elements, genes, etc). Transposable elements make up 45% of the human genome (for a review, see Zhou W. et al., 2020). Therefore, the GDMR mainly represents the stable fraction of the DNA methylation of the genome. Indeed, the differentially methylated regions over cell development or organs represent only a small fraction of the CpG of the genome.

The time series analysis performed in the present study indicated that GDMR of the blood was influenced by the date of sampling, a possible combined effect of age and season, but only slight GDMR changes were observed over time and the average GDMR was around 80%. Aging has been reported to have a profound effect on DNA methylation in many tissues and cell types (Rakyan et al., 2010; Teschendorff et al., 2010; Horvath, 2013; Issa, 2014). In sheep, like in other species, an epigenetic clock has been described that is capable of estimating chronological age and of detecting accelerated rates of aging (Sugrue et al., 2021). However in our study, the time series only covered the first 6 months of life of the lambs and this was probably too short to detect a major effect of aging on GDMR. Therefore, the date of sampling effect is hypothesized to be more related to environmental changes as the circadian cycle or the season. To better understand this time effect, we explored the influence of the complete blood count over time on blood GDMR. Indeed, Dopico et al. (2015) showed the influence of the circadian cycle on the cellular composition of blood in humans. Additionally, Goldinger et al. (2015) suggested that temperature is a major factor driving seasonal variations of this blood composition. Similarly, we showed that the blood count was influenced by the date of sampling and thus probably by the season since the first blood sample was taken at the beginning of autumn and the last at the end of winter. However, among the counts of each type of white cell, GDMR of the blood was only influenced by the neutrophil count. As the model with the date of sampling statistically fit the data better than the model with the neutrophil count, we hypothesize that the effect of sampling date may include not only changes in blood count, but also additional environmental effects influenced by time that we were unable to identify and quantify.

We first hypothesized that blood GDMR may be representative of GDMR of somatic and reproductive tissues. In our study, GDMR of somatic tissues was on average 70%, but with marked variations depending on the tissue. The GDMR of blood was higher than that of somatic tissue (whatever the tissue considered). Even if significant, all the correlations were lower than 0.63 (highest value found between ovary and blood at 3 months) and thus were unlikely to reliably predict the GDMR of one tissue from the value of another one. Thus, the use of GDMR of blood as an indicator of GDMR in less accessible tissue is questionable. Variations in GDMR of blood (CV of 2.6% within or between animals) are probably too small in our experimental animals and too close to the CV of the assay (1.4%) to establish a correlation with the GDMR measured in another tissue. Nevertheless, the present findings in sheep are consistent with those obtained in different bovine somatic tissues. Most somatic tissue DNA samples in cattle had average methylation levels of 70–80% (Zhou Y. et al., 2020). To understand the variability of DNA methylation across cattle tissues and its regulation of gene expression, Zhou Y. et al. (2020) profiled the cattle DNA methylomes in 16 major tissues using the whole genome bisulfite sequencing (WGBS) method. However, it should be kept in mind that the GDMR values obtained by WGBS are often slightly higher than the values obtained using the LUMA technology. For example, in ovine muscle, Fan et al. (2020) analyzed the DNA methylation profiles in Hu sheep muscle (an endemic Chinese sheep breed) at two key developmental stages (110-days fetuses and two-year-old adults) using WGBS technology. They found genome-wide methylated cytosine (mC) levels for CG of 88.87 ± 0.67% in fetal samples and 85.33 ± 0.95% in adult samples. In our study using the same species but lambs of different ages, the GDMR measured in muscle was lower (67.69 ± 5.71%). In sheep ovaries, Zhang et al. (2017) found an overall genome-wide methylated cytosine level of 89.78% for CpG using WGBS, whereas we found a value of 71.39 ± 2.56% using the LUMA technology. Whereas WGBS accounts for all the CpG of the genome, the LUMA technology only considers the CpG located at the CCGG restriction site. In silico, we counted 5 994 593 CCGG sites on the ovine reference genome, which gives a reliable approximation of the methylated cytosine (mC) for CG of the entire genome but may explain the lower values found with the LUMA technology than with WGBS.

In the female tissues of the reproductive axis (ovary, uterus, and oviduct), GDMR was around 70% and corresponded to the average values obtained for the other somatic tissues. In contrast, the three male tissues (testis, epididymis, and seminal vesicle) had a lower average GDMR of around 60%. It would be interesting to compare this observation with the significant effect of sex in blood showing lower GDMR in males, but we found no similar data in the literature with which to compare our results to interpret the difference between sexes. Sex hormones could be involved in this phenomenon. In sheep, it has been shown that castration feminizes parts of the epigenome and delays epigenetic aging (Sugrue et al., 2021). Comparison of intact and castrated males would allow us to identify androgen-dependent age-associated methylation changes that affect known targets of sex hormone pathways and hormone binding transcription factors in castrated sheep. In rodents, a primary effect of gonadal steroids in the highly sexually-dimorphic preoptic area (brain region) has been shown to reduce the activity of DNA methyltransferase (Dnmt), thereby decreasing DNA methylation and releasing masculinizing genes from epigenetic repression (Nugent et al., 2015). In this brain area, females had higher levels of methylation (measured by WGBS) with significantly more fully methylated CpG sites than males. In humans, the link between reproductive hormones and DNA methylation has also been described in hormonal therapy of cancer. Indeed, antiandrogen medication given to patient with prostate cancer increased *DNMT3A* and *DNMT3B* expression (Gravina et al., 2011). In the human endometrium, *DNMT* mRNA levels change during the menstrual cycle, and *DNMT3A* and *DNMT3B* mRNAs can be regulated by female sex steroid hormones in endometrial stromal cells (Yamagata et al., 2009). If sex hormones do indeed influence GDMR, a further study focusing on the time around puberty (starting from 6 months of age in sheep) would be interesting.

Investigation of GDMR of the blood in several breeds of sheep evidenced a breed specific effect mainly of the Blackbelly breed, consequently, to limit environmental effects, Blackbelly lambs were bred at the same time as Charollais and Romane lambs in the same environmental conditions. This significant influence of the breed on GDMR thus certainly reflects the influence of different genetic backgrounds. Indeed, Blackbelly sheep are well adapted to a semi-arid tropical environment (high temperature and humidity, but also to extended drought), in contrast to the Romane and Charollais breeds that are more adapted to temperate climates and different farming systems (free range, semi-free range, use of sheepfolds). The three breeds thus have different abilities to adapt to and survive in different conditions. In a previous study performed on around 700 Romane lambs reared outdoors (Hazard et al., 2020), the average GDMR of the blood was lower (around 70%) than the GDMR found in the present study (around 80%). The radically different farming system (entirely indoors vs. entirely outdoors) may partly explain the observed difference. As shown previously (Hazard et al., 2020), GDMR has a heritability of 0.20 ± 0.05 and could thus be genetically selected. DNA methylation is associated with repression of transposable elements and acts to limit their genotoxic potential. If GDMR is reduced by genetic selection, it could lead to instability of transposable elements in the genome. However, this genome plasticity could help animals adapt to changing production systems and environment. Kooke et al. (2015) showed that experimentally induced DNA hypomethylation of plants rendered them more sensitive to environmental variation and more flexible in their responses. Thus, the lower GDMR of the blood in lambs reared in extensive conditions than the GDMR level in indoor conditions may help improve sheep adaptation to a harsh environment.

## Conclusion

We explored the GDMR phenotype and investigated its variability in several tissues in different breeds of sheep reared in the same environment. The GDMR measured in blood was higher than that measured in somatic tissue whatever the tissue considered, and none of the significant correlations found was strong enough to predict a link between GDMR in different tissues. Thus, the use of GDMR of the blood as a proxy of GDMR in less accessible tissues is probably not appropriate. However, we evidenced a significant influence of the breed on GDMR, certainly reflecting the influence of different genetic backgrounds and raising questions about relationships between GDMR and the ability of these breeds of sheep to adapt to contrasting environments. Further studies will be conducted by creating genetically divergent lines for GDMR of the blood to investigate the consequences of high versus low levels of GDMR of the blood for several phenotypes including adaptive traits.

## Data Availability

The raw data supporting the conclusion of this article will be made available by the authors, without undue reservation.

## References

[B1] Dopico X. C. Evangelou M. Ferreira R. C. Guo H. Pekalski M. L. Smyth D. J. (2015). Widespread Seasonal Gene Expression Reveals Annual Differences in Human Immunity and Physiology. Nat. Commun. 6, 7000. 10.1038/ncomms8000 25965853 PMC4432600

[B2] Ehrlich M. Gama-Sosa M. A. Huang L.-H. Midgett R. M. Kuo K. C. McCune R. A. (1982). Amount and Distribution of 5-methylcytosine in Human DNA from Different Types of Tissues or Cells. Nucl. Acids Res. 10, 2709–2721. 10.1093/nar/10.8.2709 7079182 PMC320645

[B3] Fan Y. Liang Y. Deng K. Zhang Z. Zhang G. Zhang Y. (2020). Analysis of DNA Methylation Profiles during Sheep Skeletal Muscle Development Using Whole-Genome Bisulfite Sequencing. BMC Genomics 21, 327. 10.1186/s12864-020-6751-5 32349667 PMC7191724

[B4] Goddard M. E. Hayes B. J. Meuwissen T. H. E. (2010). Genomic Selection in Livestock Populations. Genet. Res. 92, 413–421. 10.1017/S0016672310000613 21429272

[B5] Goddard M. E. Whitelaw E. (2014). The Use of Epigenetic Phenomena for the Improvement of Sheep and Cattle. Front. Genet. 5, 247. 10.3389/fgene.2014.00247 25191337 PMC4139735

[B6] Goldinger A. Shakhbazov K. Henders A. K. McRae A. F. Montgomery G. W. Powell J. E. (2015). Seasonal Effects on Gene Expression. PLoS ONE 10, e0126995. 10.1371/journal.pone.0126995 26023781 PMC4449160

[B7] Gravina G. L. Marampon F. Piccolella M. Motta M. Ventura L. Pomante R. (2011). Hormonal Therapy Promotes Hormone-Resistant Phenotype by Increasing DNMT Activity and Expression in Prostate Cancer Models. Endocrinology 152, 4550–4561. 10.1210/en.2011-1056 21990314 PMC3230051

[B8] Hazard D. Plisson-Petit F. Moreno-Romieux C. Fabre S. Drouilhet L. (2020). Genetic Determinism Exists for the Global DNA Methylation Rate in Sheep. Front. Genet. 11, 1717. 10.3389/fgene.2020.616960 PMC7786236 33424937

[B9] Horvath S. (2013). DNA Methylation Age of Human Tissues and Cell Types. Genome Biol. 14, R115. 10.1186/gb-2013-14-10-r115 24138928 PMC4015143

[B10] Issa J.-P. (2014). Aging and Epigenetic Drift: a Vicious Cycle. J. Clin. Invest. 124, 24–29. 10.1172/JCI69735 24382386 PMC3871228

[B11] Johannes F. Porcher E. Teixeira F. K. Saliba-Colombani V. Simon M. Agier N. (2009). Assessing the Impact of Transgenerational Epigenetic Variation on Complex Traits. Plos Genet. 5, e1000530. 10.1371/journal.pgen.1000530 19557164 PMC2696037

[B12] Karimi M. Johansson S. Ekström T. J. (2006a). Using LUMA: a Luminometric-Based Assay for Global DNA-Methylation. Epigenetics 1, 46–49. 10.4161/epi.1.1.2587 17998810

[B13] Karimi M. Johansson S. Stach D. Corcoran M. Grandér D. Schalling M. (2006b). LUMA (LUminometric Methylation Assay)-A High Throughput Method to the Analysis of Genomic DNA Methylation. Exp. Cel Res. 312, 1989–1995. 10.1016/j.yexcr.2006.03.006 16624287

[B14] Kijas J. W. Lenstra J. A. Hayes B. Boitard S. Porto Neto L. R. San Cristobal M. (2012). Genome-Wide Analysis of the World's Sheep Breeds Reveals High Levels of Historic Mixture and Strong Recent Selection. Plos Biol. 10, e1001258. 10.1371/journal.pbio.1001258 22346734 PMC3274507

[B15] Kooke R. Johannes F. Wardenaar R. Becker F. Etcheverry M. Colot V. (2015). Epigenetic Basis of Morphological Variation and Phenotypic Plasticity in *Arabidopsis thaliana* . The Plant Cell 27, 337–348. 10.1105/tpc.114.133025 25670769 PMC4456930

[B17] Lander E. S. Linton L. M. Birren B. Nusbaum C. Zody M. C. Baldwin J. (2001). Initial Sequencing and Analysis of the Human Genome. Nature 409, 860–921. 10.1038/35057062 11237011

[B18] Latzel V. Allan E. Bortolini Silveira A. Colot V. Fischer M. Bossdorf O. (2013). Epigenetic Diversity Increases the Productivity and Stability of Plant Populations. Nat. Commun. 4, 2875. 10.1038/ncomms3875 24285012

[B19] Marie H. Laurent G. Sophie M. De Hubert R. Coralie D.-B. Verrier É. (2003). Genetic Variability of Six French Meat Sheep Breeds in Relation to Their Genetic Management. Genet. Sel Evol. 35, 637. 10.1186/1297-9686-35-7-637 14604512 PMC2698003

[B20] Murdoch B. M. Murdoch G. K. Greenwood S. McKay S. (2016). Nutritional Influence on Epigenetic Marks and Effect on Livestock Production. Front. Genet. 7, 182. 10.3389/fgene.2016.00182 27822224 PMC5075561

[B21] Nugent B. M. Wright C. L. Shetty A. C. Hodes G. E. Lenz K. M. Mahurkar A. (2015). Brain Feminization Requires Active Repression of Masculinization via DNA Methylation. Nat. Neurosci. 18, 690–697. 10.1038/nn.3988 25821913 PMC4519828

[B22] Rakyan V. K. Down T. A. Maslau S. Andrew T. Yang T.-P. Beyan H. (2010). Human Aging-Associated DNA Hypermethylation Occurs Preferentially at Bivalent Chromatin Domains. Genome Res. 20, 434–439. 10.1101/gr.103101.109 20219945 PMC2847746

[B23] Ricordeau G. Tchamitchian L. Brunel J. C. Nguyen T. C. François D. (1992). La gestion des populations : La race ovine INRA 401 : un exemple de souche synthétique. INRA Prod. Anim. 5, 255–262. 10.20870/productions-animales.1992.5.hs.4300

[B24] Rochus C. M. Tortereau F. Plisson-Petit F. Restoux G. Moreno-Romieux C. Tosser-Klopp G. (2018). Revealing the Selection History of Adaptive Loci Using Genome-wide Scans for Selection: an Example from Domestic Sheep. BMC Genomics 19, 71. 10.1186/s12864-018-4447-x 29357834 PMC5778797

[B25] Roussot O. Feve K. Plisson-Petit F. Pitel F. Faure J.-M. Beaumont C. (2003). AFLP Linkage Map of the Japanese Quail Coturnix japonica. Genet. Sel Evol. 35, 559. 10.1186/1297-9686-35-6-559 12939205 PMC2697981

[B26] Sallé G. Jacquiet P. Gruner L. Cortet J. Sauvé C. Prévot F. (2012). A Genome Scan for QTL Affecting Resistance to Haemonchus contortus in Sheep1. J. Anim. Sci. 90, 4690–4705. 10.2527/jas.2012-5121 22767094

[B27] SAS (2008). Statistical Analysis Systems Institute. Version 9.1. Cary, North Carolina, USA: SAS Institute Inc.

[B28] Sinclair K. D. Allegrucci C. Singh R. Gardner D. S. Sebastian S. Bispham J. (2007). DNA Methylation, Insulin Resistance, and Blood Pressure in Offspring Determined by Maternal Periconceptional B Vitamin and Methionine Status. Proc. Natl. Acad. Sci. 104, 19351–19356. 10.1073/pnas.0707258104 18042717 PMC2148293

[B29] Spangler G. L. Rosen B. D. Ilori M. B. Hanotte O. Kim E.-S. Sonstegard T. S. (2017). Whole Genome Structural Analysis of Caribbean Hair Sheep Reveals Quantitative Link to West African Ancestry. PLoS ONE 12, e0179021. 10.1371/journal.pone.0179021 28662044 PMC5490989

[B30] Stadler M. B. Murr R. Burger L. Ivanek R. Lienert F. Schöler A. (2011). DNA-binding Factors Shape the Mouse Methylome at Distal Regulatory Regions. Nature 480, 490–495. 10.1038/nature10716 22170606

[B31] Sugrue V. J. Zoller J. A. Narayan P. Lu A. T. Ortega-Recalde O. J. Grant M. J. (2021). Castration Delays Epigenetic Aging and Feminizes DNA Methylation at Androgen-Regulated Loci. eLife 10, e64932. 10.7554/eLife.64932 34227937 PMC8260231

[B32] Suzuki M. M. Bird A. (2008). DNA Methylation Landscapes: Provocative Insights from Epigenomics. Nat. Rev. Genet. 9, 465–476. 10.1038/nrg2341 18463664

[B33] Teschendorff A. E. Menon U. Gentry-Maharaj A. Ramus S. J. Weisenberger D. J. Shen H. (2010). Age-dependent DNA Methylation of Genes that Are Suppressed in Stem Cells Is a Hallmark of Cancer. Genome Res. 20, 440–446. 10.1101/gr.103606.109 20219944 PMC2847747

[B34] Venter J. C. Adams M. D. Myers E. W. Li P. W. Mural R. J. Sutton G. G. (2001). The Sequence of the Human Genome. Science 291, 1304–1351. 10.1126/science.1058040 11181995

[B35] Weaver I. C. G. Cervoni N. Champagne F. A. D'Alessio A. C. Sharma S. Seckl J. R. (2004). Epigenetic Programming by Maternal Behavior. Nat. Neurosci. 7, 847–854. 10.1038/nn1276 15220929

[B36] Yamagata Y. Asada H. Tamura I. Lee L. Maekawa R. Taniguchi K. (2009). DNA Methyltransferase Expression in the Human Endometrium: Down-Regulation by Progesterone and Estrogen. Hum. Reprod. 24, 1126–1132. 10.1093/humrep/dep015 19202141

[B37] Zhang Y. Li F. Feng X. Yang H. Zhu A. Pang J. (2017). Genome-wide Analysis of DNA Methylation Profiles on Sheep Ovaries Associated with Prolificacy Using Whole-Genome Bisulfite Sequencing. BMC Genomics 18, 759. 10.1186/s12864-017-4068-9 28969601 PMC5625832

[B38] Zhou W. Liang G. Molloy P. L. Jones P. A. (2020). DNA Methylation Enables Transposable Element-Driven Genome Expansion. Proc. Natl. Acad. Sci. USA 117, 19359–19366. 10.1073/pnas.1921719117 32719115 PMC7431005

[B39] Zhou Y. Liu S. Hu Y. Fang L. Gao Y. Xia H. (2020). Comparative Whole Genome DNA Methylation Profiling across Cattle Tissues Reveals Global and Tissue-specific Methylation Patterns. BMC Biol. 18, 85. 10.1186/s12915-020-00793-5 32631327 PMC7339546

