# Supplementary material for: Variability in Global DNA Methylation Rate Across Tissues and Over Time in Sheep
Source: Front Genet. 2022 Mar 11;13:791283. doi: 10.3389/fgene.2022.791283 (PMC8961874; doi:10.3389/fgene.2022.791283)
Supplement: Supplementary file 1 [file Table1.DOCX]

**Supplementary Table S1**: Sex, litter size and breed of lambs.

| Animal | Sex | Litter size | Breed | Tissues collection |
| --- | --- | --- | --- | --- |
| Lamb 1 | M | 2 | Blackbelly | * |
| Lamb 2 | M | 2 | Blackbelly | * |
| Lamb 3 | M | 2 | Blackbelly |  |
| Lamb 4 | M | 2 | Blackbelly | * |
| Lamb 5 | M | 1 | Blackbelly | * |
| Lamb 6 | M | 2 | Blackbelly |  |
| Lamb 7 | M | 1 | Blackbelly | * |
| Lamb 8 | F | 3 | Blackbelly |  |
| Lamb 9 | M | 2 | Blackbelly | * |
| Lamb 10 | M | 2 | Blackbelly |  |
| Lamb 11 | M | 2 | Blackbelly | * |
| Lamb 12 | M | 2 | Blackbelly | * |
| Lamb 13 | M | 2 | Blackbelly |  |
| Lamb 14 | F | 2 | Blackbelly |  |
| Lamb 15 | M | 2 | Blackbelly |  |
| Lamb 16 | M | 2 | Blackbelly |  |
| Lamb 17 | F | 2 | Blackbelly | * |
| Lamb 18 | F | 1 | Blackbelly | * |
| Lamb 19 | F | 3 | Blackbelly | * |
| Lamb 20 | F | 2 | Blackbelly | * |
| Lamb 21 | F | 2 | Blackbelly | * |
| Lamb 22 | F | 2 | Blackbelly | * |
| Lamb 23 | F | 2 | Blackbelly | * |
| Lamb 24 | F | 1 | Charollais |  |
| Lamb 25 | F | 1 | Charollais |  |
| Lamb 26 | F | 2 | Charollais |  |
| Lamb 27 | F | 2 | Charollais |  |
| Lamb 28 | F | 1 | Charollais |  |
| Lamb 29 | M | 2 | Charollais |  |
| Lamb 30 | M | 2 | Charollais |  |
| Lamb 31 | F | 2 | Charollais |  |
| Lamb 32 | F | 2 | Charollais |  |
| Lamb 33 | F | 2 | Charollais |  |
| Lamb 34 | F | 2 | Charollais |  |
| Lamb 35 | F | 1 | Charollais |  |
| Lamb 36 | M | 3 | Romane | * |
| Lamb 37 | M | 2 | Romane | * |
| Lamb 38 | M | 2 | Romane | * |
| Lamb 39 | M | 2 | Romane | ***** |
| Lamb 40 | M | 2 | Romane | ***** |
| Lamb 41 | M | 1 | Romane | ***** |
| Lamb 42 | M | 1 | Romane | ***** |
| Lamb 43 | F | 2 | Romane |  |
| Lamb 44 | M | 2 | Romane |  |
| Lamb 45 | M | 2 | Romane | ***** |
| Lamb 46 | M | 2 | Romane |  |
| Lamb 47 | M | 2 | Romane |  |
| Lamb 48 | F | 2 | Romane |  |
| Lamb 49 | F | 2 | Romane |  |
| Lamb 50 | F | 2 | Romane |  |
| Lamb 51 | F | 2 | Romane | * |
| Lamb 52 | F | 2 | Romane | * |
| Lamb 53 | F | 2 | Romane | * |
| Lamb 54 | F | 2 | Romane | ***** |
| Lamb 55 | F | 2 | Romane | ***** |
| Lamb 56 | F | 2 | Romane | ***** |
| Lamb 57 | F | 2 | Romane |  |
| Lamb 58 | F | 2 | Romane | ***** |
| Lamb 59 | M | 1 | Romane |  |

* : the corresponding animal was slaughtered for tissue collection.
