# Supplementary material for: Variability in Global DNA Methylation Rate Across Tissues and Over Time in Sheep
Source: Front Genet. 2022 Mar 11;13:791283. doi: 10.3389/fgene.2022.791283 (PMC8961874; doi:10.3389/fgene.2022.791283)
Supplement: Supplementary file 2 [file Table2.DOCX]

**Supplementary Table S2**: Sampling sites of the tissue collection.

| Tissue | Sampling site |
| --- | --- |
| frontal lobe | partial, ventral part |
| pituitary gland | entire pituitary |
| heart | partial, cardiac muscle tip |
| lung | partial, larger ventral lobe |
| sub cutaneous adipose tissue | partial, abdonimal |
| perirenal adipose tissue | partial, left kidney |
| muscle | partial, inner left thigh |
| liver | partial, ventral extremity |
| spleen | partial, ventral extremity |
| adrenal gland | entire left and right adrenals |
| medulla kidney | partial, inner clear area of the left kidney |
| cortical kidney | partial, outer colored area of the left kidney |
| ovary | entire, left ovary |
| oviduct | entire, left oviduct |
| uterus | partial, median part of the left horn |
| testis | partial, median part of the left testis, without albuginea |
| epididymis | entire left epididymis (caput, body, cauda) |
| seminal vesicle | entire seminal vesicle |
