# Supplementary material for: Variability in Global DNA Methylation Rate Across Tissues and Over Time in Sheep
Source: Front Genet. 2022 Mar 11;13:791283. doi: 10.3389/fgene.2022.791283 (PMC8961874; doi:10.3389/fgene.2022.791283)
Supplement: Supplementary file 3 [file Table3.DOCX]

|  | Birth blood | 1 month blood | 2 months blood | 3 months blood | 4 months blood | 5 months blood | epididymis | testis | seminal vesicle | uterus | oviduct | ovary | frontal lobe | muscle | spleen | lung | cortical kidney | medulla kidney | adrenal gland | adipose tissu | heart | liver | perirenal adi. tis. | pituitary gland |
| --- | --- | --- | --- | --- | --- | --- | --- | --- | --- | --- | --- | --- | --- | --- | --- | --- | --- | --- | --- | --- | --- | --- | --- | --- |
| Birth blood | 1 | -0.15 | -0.02 | -0.18 | -0.23 | 0.07 | -0.03 | 0.23 | -0.09 | 0.33 | 0.28 | -0.42 | 0.07 | -0.03 | 0.20 | 0.01 | -0.09 | 0.17 | 0.52 | 0.36 | -0.12 | 0.23 | 0.15 | -0.11 |
|  |  | 0.44 | 0.92 | 0.34 | 0.26 | 0.70 | 0.91 | 0.48 | 0.75 | 0.25 | 0.33 | 0.14 | 0.72 | 0.87 | 0.32 | 0.96 | 0.64 | 0.37 | 0.004 | 0.07 | 0.54 | 0.23 | 0.45 | 0.55 |
|  | 29 | 29 | 29 | 29 | 26 | 29 | 15 | 12 | 15 | 14 | 14 | 14 | 29 | 29 | 28 | 28 | 28 | 29 | 29 | 29 | 28 | 28 | 29 | 29 |
| 1 month blood |  | 1 | 0.43 | 0.17 | 0.14 | 0.30 | 0.20 | -0.05 | 0.42 | -0.12 | -0.27 | 0.01 | 0.53 | 0.19 | -0.17 | 0.11 | -0.16 | 0.22 | 0.19 | -0.09 | -0.26 | -0.05 | 0.02 | 0.15 |
|  |  |  | 0.02 | 0.37 | 0.49 | 0.11 | 0.45 | 0.87 | 0.10 | 0.69 | 0.35 | 0.98 | 0.002 | 0.31 | 0.38 | 0.56 | 0.41 | 0.23 | 0.30 | 0.62 | 0.17 | 0.80 | 0.92 | 0.42 |
|  |  | 30 | 30 | 30 | 27 | 30 | 16 | 13 | 16 | 14 | 14 | 14 | 30 | 30 | 29 | 29 | 29 | 30 | 30 | 30 | 29 | 29 | 30 | 30 |
| 2 months blood |  |  | 1 | 0.04 | -0.15 | 0.001 | 0.01 | 0.20 | -0.13 | 0.11 | -0.01 | -0.42 | 0.38 | -0.06 | 0.19 | -0.03 | -0.27 | 0.12 | 0.22 | -0.01 | 0.15 | -0.22 | -0.36 | 0.04 |
|  |  |  |  | 0.82 | 0.46 | 0.99 | 0.97 | 0.52 | 0.64 | 0.71 | 0.96 | 0.13 | 0.04 | 0.75 | 0.32 | 0.87 | 0.16 | 0.52 | 0.24 | 0.97 | 0.44 | 0.25 | 0.05 | 0.84 |
|  |  |  | 30 | 30 | 27 | 30 | 16 | 13 | 16 | 14 | 14 | 14 | 30 | 30 | 29 | 29 | 29 | 30 | 30 | 30 | 29 | 29 | 30 | 30 |
| 3 months blood |  |  |  | 1 | 0.03 | 0.19 | -0.14 | -0.06 | -0.02 | -0.23 | -0.31 | 0.63 | 0.21 | -0.05 | -0.13 | 0.10 | -0.29 | 0.04 | -0.29 | 0.11 | -0.17 | -0.15 | -0.15 | -0.10 |
|  |  |  |  |  | 0.86 | 0.32 | 0.60 | 0.86 | 0.93 | 0.42 | 0.29 | 0.02 | 0.27 | 0.80 | 0.49 | 0.60 | 0.13 | 0.83 | 0.12 | 0.58 | 0.39 | 0.44 | 0.42 | 0.58 |
|  |  |  |  | 30 | 27 | 30 | 16 | 13 | 16 | 14 | 14 | 14 | 30 | 30 | 29 | 29 | 29 | 30 | 30 | 30 | 29 | 29 | 30 | 30 |
| 4 months blood |  |  |  |  | 1 | 0.48 | 0.07 | 0.17 | 0.11 | 0.09 | 0.001 | -0.22 | 0.25 | 0.11 | -0.14 | 0.33 | -0.11 | 0.35 | -0.10 | 0.01 | 0.04 | 0.46 | 0.12 | -0.06 |
|  |  |  |  |  |  | 0.01 | 0.82 | 0.59 | 0.71 | 0.76 | 0.99 | 0.45 | 0.20 | 0.59 | 0.49 | 0.10 | 0.58 | 0.08 | 0.61 | 0.96 | 0.86 | 0.02 | 0.57 | 0.77 |
|  |  |  |  |  | 27 | 27 | 13 | 12 | 13 | 14 | 14 | 14 | 27 | 27 | 26 | 26 | 26 | 27 | 27 | 27 | 26 | 26 | 27 | 27 |
| 5 months blood |  |  |  |  |  | 1 | 0.37 | 0.18 | 0.10 | 0.55 | 0.14 | 0.04 | 0.31 | 0.07 | -0.23 | 0.33 | -0.12 | 0.32 | -0.04 | 0.03 | 0.09 | 0.26 | 0.23 | 0.07 |
|  |  |  |  |  |  |  | 0.16 | 0.55 | 0.71 | 0.04 | 0.64 | 0.90 | 0.09 | 0.71 | 0.22 | 0.08 | 0.54 | 0.08 | 0.83 | 0.86 | 0.64 | 0.17 | 0.22 | 0.71 |
|  |  |  |  |  |  | 30 | 16 | 13 | 16 | 14 | 14 | 14 | 30 | 30 | 29 | 29 | 29 | 30 | 30 | 30 | 29 | 29 | 30 | 30 |
| epididymis |  |  |  |  |  |  | 1 | -0.06 | 0.17 | . | . | . | -0.005 | 0.06 | -0.35 | 0.41 | 0.37 | 0.02 | -0.32 | -0.23 | 0.26 | -0.29 | -0.14 | -0.17 |
|  |  |  |  |  |  |  |  | 0.85 | 0.54 |  |  |  | 0.99 | 0.84 | 0.20 | 0.13 | 0.18 | 0.92 | 0.23 | 0.39 | 0.33 | 0.29 | 0.60 | 0.52 |
|  |  |  |  |  |  |  | 16 | 13 | 16 |  |  |  | 16 | 16 | 15 | 15 | 15 | 16 | 16 | 16 | 16 | 15 | 16 | 16 |
| testis |  |  |  |  |  |  |  | 1 | 0.47 | . | . | . | 0.28 | 0.55 | 0.12 | 0.23 | -0.36 | 0.46 | -0.14 | -0.23 | 0.52 | 0.33 | -0.09 | 0.34 |
|  |  |  |  |  |  |  |  |  | 0.10 |  |  |  | 0.36 | 0.05 | 0.72 | 0.46 | 0.22 | 0.11 | 0.66 | 0.45 | 0.07 | 0.30 | 0.77 | 0.26 |
|  |  |  |  |  |  |  |  | 13 | 13 |  |  |  | 13 | 13 | 12 | 12 | 13 | 13 | 13 | 13 | 13 | 12 | 13 | 13 |

**Supplementary Table S3** : Tissue-to-tissue GDMR correlation

|  | Birth blood | 1 month blood | 2 month blood | 3 month blood | 4 month blood | 5 month blood | epididymis | testis | seminal vesicle | uterus | oviduct | ovary | frontal lobe | muscle | spleen | lung | cortical kidney | medulla kidney | adrenal gland | adipose tissu | heart | liver | perirenal adi. tis. | pituitary gland |
| --- | --- | --- | --- | --- | --- | --- | --- | --- | --- | --- | --- | --- | --- | --- | --- | --- | --- | --- | --- | --- | --- | --- | --- | --- |
| seminal vesicle |  |  |  |  |  |  |  |  | 1 | . | . | . | 0.36 | 0.36 | -0.03 | 0.12 | -0.38 | 0.55 | -0.19 | -0.26 | -0.24 | -0.09 | 0.11 | 0.36 |
|  |  |  |  |  |  |  |  |  |  |  |  |  | 0.17 | 0.17 | 0.92 | 0.68 | 0.16 | 0.03 | 0.49 | 0.33 | 0.36 | 0.75 | 0.68 | 0.17 |
|  |  |  |  |  |  |  |  |  | 16 |  |  |  | 16 | 16 | 15 | 15 | 15 | 16 | 16 | 16 | 16 | 15 | 16 | 16 |
| uterus |  |  |  |  |  |  |  |  |  | 1 | 0.11 | -0.23 | 0.16 | -0.10 | 0.41 | 0.09 | -0.13 | 0.18 | 0.11 | -0.05 | 0.24 | 0.07 | 0.17 | 0.34 |
|  |  |  |  |  |  |  |  |  |  |  | 0.70 | 0.42 | 0.60 | 0.74 | 0.14 | 0.77 | 0.65 | 0.55 | 0.71 | 0.85 | 0.43 | 0.81 | 0.56 | 0.23 |
|  |  |  |  |  |  |  |  |  |  | 14 | 14 | 14 | 14 | 14 | 14 | 14 | 14 | 14 | 14 | 14 | 13 | 14 | 14 | 14 |
| oviduct |  |  |  |  |  |  |  |  |  |  | 1 | 0.07 | -0.09 | -0.48 | 0.19 | -0.03 | -0.29 | 0.18 | -0.15 | 0.26 | 0.15 | 0.09 | -0.24 | -0.07 |
|  |  |  |  |  |  |  |  |  |  |  |  | 0.82 | 0.77 | 0.08 | 0.52 | 0.91 | 0.31 | 0.53 | 0.62 | 0.36 | 0.63 | 0.75 | 0.42 | 0.83 |
|  |  |  |  |  |  |  |  |  |  |  | 14 | 14 | 14 | 14 | 14 | 14 | 14 | 14 | 14 | 14 | 13 | 14 | 14 | 14 |
| ovary |  |  |  |  |  |  |  |  |  |  |  | 1 | -0.22 | -0.24 | -0.34 | -0.14 | 0.16 | -0.42 | -0.57 | 0.05 | -0.42 | -0.17 | -0.002 | -0.10 |
|  |  |  |  |  |  |  |  |  |  |  |  |  | 0.46 | 0.40 | 0.23 | 0.64 | 0.59 | 0.13 | 0.04 | 0.87 | 0.15 | 0.55 | 0.99 | 0.73 |
|  |  |  |  |  |  |  |  |  |  |  |  | 14 | 14 | 14 | 14 | 14 | 14 | 14 | 14 | 14 | 13 | 14 | 14 | 14 |
| frontal lobe |  |  |  |  |  |  |  |  |  |  |  |  | 1 | -0.05 | -0.12 | 0.45 | -0.26 | 0.18 | 0.04 | 0.32 | -0.004 | 0.30 | -0.06 | 0.13 |
|  |  |  |  |  |  |  |  |  |  |  |  |  |  | 0.81 | 0.55 | 0.01 | 0.17 | 0.35 | 0.84 | 0.09 | 0.98 | 0.11 | 0.75 | 0.49 |
|  |  |  |  |  |  |  |  |  |  |  |  |  | 30 | 30 | 29 | 29 | 29 | 30 | 30 | 30 | 29 | 29 | 30 | 30 |
| muscle |  |  |  |  |  |  |  |  |  |  |  |  |  | 1 | -0.12 | 0.03 | -0.19 | 0.06 | -0.12 | 0.01 | -0.06 | 0.11 | 0.22 | 0.17 |
|  |  |  |  |  |  |  |  |  |  |  |  |  |  |  | 0.55 | 0.87 | 0.33 | 0.76 | 0.54 | 0.97 | 0.74 | 0.56 | 0.25 | 0.38 |
|  |  |  |  |  |  |  |  |  |  |  |  |  |  | 30 | 29 | 29 | 29 | 30 | 30 | 30 | 29 | 29 | 30 | 30 |
| spleen |  |  |  |  |  |  |  |  |  |  |  |  |  |  | 1 | -0.14 | -0.09 | 0.09 | 0.02 | -0.07 | 0.26 | 0.07 | -0.33 | 0.04 |
|  |  |  |  |  |  |  |  |  |  |  |  |  |  |  |  | 0.47 | 0.66 | 0.66 | 0.90 | 0.73 | 0.18 | 0.73 | 0.08 | 0.84 |
|  |  |  |  |  |  |  |  |  |  |  |  |  |  |  | 29 | 28 | 28 | 29 | 29 | 29 | 28 | 28 | 29 | 29 |
| lung |  |  |  |  |  |  |  |  |  |  |  |  |  |  |  | 1 | -0.12 | 0.12 | -0.33 | 0.18 | 0.21 | 0.38 | 0.06 | -0.34 |
|  |  |  |  |  |  |  |  |  |  |  |  |  |  |  |  |  | 0.54 | 0.55 | 0.08 | 0.34 | 0.30 | 0.05 | 0.77 | 0.07 |
|  |  |  |  |  |  |  |  |  |  |  |  |  |  |  |  | 29 | 28 | 29 | 29 | 29 | 28 | 28 | 29 | 29 |

|  | Birth blood | 1 month blood | 2 month blood | 3 month blood | 4 month blood | 5 month blood | epididymis | testis | seminal vesicle | uterus | oviduct | ovary | frontal lobe | muscle | spleen | lung | cortical kidney | medulla kidney | adrenal gland | adipose tissu | heart | liver | perirenal adi. tis. | pituitary gland |
| --- | --- | --- | --- | --- | --- | --- | --- | --- | --- | --- | --- | --- | --- | --- | --- | --- | --- | --- | --- | --- | --- | --- | --- | --- |
| cortical kidney |  |  |  |  |  |  |  |  |  |  |  |  |  |  |  |  | 1 | -0.37 | 0.08 | -0.04 | 0.13 | 0.03 | 0.17 | -0.04 |
|  |  |  |  |  |  |  |  |  |  |  |  |  |  |  |  |  |  | 0.05 | 0.69 | 0.82 | 0.51 | 0.87 | 0.37 | 0.83 |
|  |  |  |  |  |  |  |  |  |  |  |  |  |  |  |  |  | 29 | 29 | 29 | 29 | 28 | 28 | 29 | 29 |
| medulla kidney |  |  |  |  |  |  |  |  |  |  |  |  |  |  |  |  |  | 1 | 0.15 | -0.23 | -0.12 | 0.29 | 0.10 | 0.45 |
|  |  |  |  |  |  |  |  |  |  |  |  |  |  |  |  |  |  |  | 0.43 | 0.22 | 0.55 | 0.13 | 0.59 | 0.01 |
|  |  |  |  |  |  |  |  |  |  |  |  |  |  |  |  |  |  | 30 | 30 | 30 | 29 | 29 | 30 | 30 |
| adrenal gland |  |  |  |  |  |  |  |  |  |  |  |  |  |  |  |  |  |  | 1 | 0.02 | -0.27 | 0.08 | 0.20 | 0.10 |
|  |  |  |  |  |  |  |  |  |  |  |  |  |  |  |  |  |  |  |  | 0.91 | 0.15 | 0.67 | 0.28 | 0.61 |
|  |  |  |  |  |  |  |  |  |  |  |  |  |  |  |  |  |  |  | 30 | 30 | 29 | 29 | 30 | 30 |
| adipose tissu |  |  |  |  |  |  |  |  |  |  |  |  |  |  |  |  |  |  |  | 1 | -0.14 | 0.15 | -0.25 | -0.26 |
|  |  |  |  |  |  |  |  |  |  |  |  |  |  |  |  |  |  |  |  |  | 0.46 | 0.44 | 0.19 | 0.16 |
|  |  |  |  |  |  |  |  |  |  |  |  |  |  |  |  |  |  |  |  | 30 | 29 | 29 | 30 | 30 |
| heart |  |  |  |  |  |  |  |  |  |  |  |  |  |  |  |  |  |  |  |  | 1 | -0.20 | -0.15 | 0.06 |
|  |  |  |  |  |  |  |  |  |  |  |  |  |  |  |  |  |  |  |  |  |  | 0.32 | 0.43 | 0.76 |
|  |  |  |  |  |  |  |  |  |  |  |  |  |  |  |  |  |  |  |  |  | 29 | 28 | 29 | 29 |
| liver |  |  |  |  |  |  |  |  |  |  |  |  |  |  |  |  |  |  |  |  |  | 1 | 0.10 | 0.01 |
|  |  |  |  |  |  |  |  |  |  |  |  |  |  |  |  |  |  |  |  |  |  |  | 0.59 | 0.94 |
|  |  |  |  |  |  |  |  |  |  |  |  |  |  |  |  |  |  |  |  |  |  | 29 | 29 | 29 |
| perirenal adi. tis. |  |  |  |  |  |  |  |  |  |  |  |  |  |  |  |  |  |  |  |  |  |  | 1 | 0.29 |
|  |  |  |  |  |  |  |  |  |  |  |  |  |  |  |  |  |  |  |  |  |  |  |  | 0.13 |
|  |  |  |  |  |  |  |  |  |  |  |  |  |  |  |  |  |  |  |  |  |  |  | 30 | 30 |

For each tissue, the first row corresponds to the correlation value, the second row to the *P* value associated and the third row to the number of samples used. perirenal adj. tis.: perirenal adipose tissue.
